# Emotion regulation in social anxiety disorder: behavioral and neural responses to three socio-emotional tasks

**DOI:** 10.1186/2045-5380-3-20

**Published:** 2013-11-04

**Authors:** Michal Ziv, Philippe R Goldin, Hooria Jazaieri, Kevin S Hahn, James J Gross

**Affiliations:** 1 Department of Psychology, Stanford University, Jordan Hall, Bldg. 420, 94305-2130 Stanford, CA, USA

**Keywords:** Social anxiety, Emotion regulation, Reappraisal, fMRI, DMPFC, Temporal dynamics

## Abstract

**Background:**

Social anxiety disorder (SAD) is thought to involve deficits in emotion regulation, and more specifically, deficits in cognitive reappraisal. However, evidence for such deficits is mixed.

**Methods:**

Using functional magnetic resonance imaging (fMRI) of blood oxygen-level dependent (BOLD) signal, we examined reappraisal-related behavioral and neural responses in 27 participants with generalized SAD and 27 healthy controls (HC) during three socio-emotional tasks: (1) looming harsh faces (Faces); (2) videotaped actors delivering social criticism (Criticism); and (3) written autobiographical negative self-beliefs (Beliefs).

**Results:**

Behaviorally, compared to HC, participants with SAD had lesser reappraisal-related reduction in negative emotion in the Beliefs task. Neurally, compared to HC, participants with SAD had lesser BOLD responses in reappraisal-related brain regions when reappraising faces, in visual and attention related regions when reappraising criticism, and in the left superior temporal gyrus when reappraising beliefs. Examination of the temporal dynamics of BOLD responses revealed late reappraisal-related increased responses in HC, compared to SAD. In addition, the dorsomedial prefrontal cortex (DMPFC), which showed reappraisal-related increased activity in both groups, had similar temporal dynamics in SAD and HC during the Faces and Criticism tasks, but greater late response increases in HC, compared to SAD, during the Beliefs task. Reappraisal-related greater late DMPFC responses were associated with greater percent reduction in negative emotion ratings in SAD patients.

**Conclusions:**

These results suggest a dysfunction of cognitive reappraisal in SAD patients, with overall reduced late brain responses in prefrontal regions, particularly when reappraising faces. Decreased late activity in the DMPFC might be associated with deficient reappraisal and greater negative reactivity.

**Trial registration:**

ClinicalTrials.gov identifier: NCT00380731

## Background

Social anxiety disorder (SAD) is characterized by heightened anxiety in a wide array of social situations [1]. It has been suggested that these elevated levels of anxiety may be due to ineffective emotion regulation [2]. Specifically, it has been suggested that patients with SAD use adaptive emotion regulation strategies, such as cognitive reappraisal, less frequently than non-anxious healthy adults [3]. In healthy individuals, cognitive reappraisal, which involves changing the meaning of a stimulus that gives rise to an emotion, can modify emotional reactions to anxiety-provoking situations, leading to greater psychological flexibility and emotional well being [4]. In SAD, deficits in cognitive reappraisal are thought to be related to difficulty in modifying the negative thoughts that arise before, during, and after social evaluative situations [5].

### Neuroimaging findings

One source of evidence regarding reappraisal in SAD comes from neuroimaging studies. To date, three studies have investigated the neural correlates of cognitive reappraisal in SAD. In the first study, Goldin and colleagues examined cognitive reappraisal of social (harsh facial expressions) and physical (violent scenes) threat in SAD patients and healthy controls. Regulation during social threat, but not physical threat, was associated with diminished recruitment of brain systems implicated in cognitive reappraisal (dorsomedial and dorsolateral prefrontal cortex (PFC)), and attention modulation (medial cuneus, posterior cingulate cortex, and parietal cortex) in SAD, compared to healthy controls [5].

In a second study by this group, using negative self-beliefs, the temporal dynamics of the BOLD response, in addition to its signal magnitude, were analyzed [3]. Findings revealed greater early activity in healthy controls during reappraisal of negative self-beliefs in brain networks implicated in reappraisal (dorsomedial, dorsolateral, and ventrolateral PFC), language (left inferior frontal gyrus), and visual (precuneus, inferior parietal) processing, while SAD patients had greater late responses in dorsomedial and ventrolateral PFC, insula, and visual processing regions. Taken together, these two studies suggest a failure to recruit reappraisal-related prefrontal regions in SAD. This differential activity of prefrontal regulatory regions in SAD has been observed in other neuroimaging studies [6-10].

The third study that explicitly addressed the neural correlates of emotion regulation in SAD, by Bruhl and colleagues [11], reported results that are discrepant with the two studies reported above. In this study, patients were instructed either to perform ‘reality checking’ or to simply anticipate and then look at negative, positive, and neutral pictures. Thus, in contrast to the first two studies, which compared patients to healthy controls, this study compared patients applying cognitive control with patients not using cognitive control. The authors found regulation-related reduced activity in emotion reactivity regions (amygdala, insula, thalamus), and in the dorsolateral PFC and cingulated cortex, with no regions with increased activity due to cognitive control.

### Explaining mixed findings

One possible explanation for the mixed findings may be the different contexts that were examined. Each of these studies focused on only one emotional probe: faces, beliefs, or non-socially specific affective pictures. However, in the SAD literature, it has been shown that the choice of emotional probes and experimental paradigms has a much larger effect on the results of fMRI investigations than is typically thought [12].

In addition to using different stimuli, another source of variability in the imaging literature is the different data analytic approaches used in each study. The majority of studies in SAD so far have used BOLD signal magnitude analyses that usually collapse across time. Only a handful of studies examined the temporal dynamics of the BOLD responses [3,13-15]. Of these studies, only one [3] has specifically focused on emotion regulation processes. Analysis of BOLD signal temporal dynamics can reveal information that might be hidden when averaging across time. It can also reveal between-group differences in brain regions that have the same averaged response among two groups, such as a pattern of early brain responses in one group, or delayed responses in the other group. Indeed, the one study examining temporal dynamics of emotion regulation processes in SAD found delayed BOLD signal onset in the dorsomedial and ventrolateral PFC regions in patients, relatively to healthy controls [3].

### The present study

The goal of the present study was to examine behavioral and fMRI BOLD responses in patients with SAD compared to healthy controls (HC) when reappraising socio-emotional stimuli. To test whether emotion dysregulation in SAD is related to a specific socio-emotional stimulus, or whether this is a more general core deficit, three contexts were compared: (1) looming harsh faces (Faces); (2) dynamic video clips of actors delivering social criticism (Criticism); and (3) written autobiographical social anxiety-related negative self-beliefs (Beliefs). To our knowledge, no study has directly tested reappraisal-related BOLD responses in patients with SAD across several distinct socio-emotional tasks that vary in both content and form.

Behaviorally, we hypothesized that, compared to HC, patients with SAD would be less successful in down regulating negative emotional reactivity when implementing cognitive reappraisal in each of the three tasks. Neurally, we hypothesized that, compared to HC, patients with SAD would have: (1) lesser BOLD responses in reappraisal-related PFC regions; and (2) delayed (that is, late) BOLD responses in reappraisal-related PFC regions. In addition to analyzing the differential and common BOLD responses separately for each task, we examined BOLD signal temporal dynamics in regions showing similar reappraisal-related increased activity in SAD and HC across all three tasks. The purpose of this analysis was to see whether there are cognitive reappraisal-related brain responses that are stimulus-independent, and whether more refined timing differences in patients and HC could be revealed using analyses of the BOLD signal temporal dynamics.

## Methods

### Participants

This study was part of a randomized controlled trial (RCT) of cognitive-behavioral therapy (CBT) for SAD, and data from participants in this RCT have been published in other baseline brain papers [3,10]. Participants included 27 (12 women) unmedicated (minimum of three months since stopping pharmacotherapy) adults who met *DSM-IV-TR*[16] criteria for primary generalized SAD and 27 (13 women) HC with no lifetime history of psychiatric disorders (Table 1). As reported in Ziv et al. [10], patients were recruited through clinician referrals and advertisements on community and online bulletin boards. Two PhD-level clinical psychologists assessed each potential participant using the Anxiety Disorders Interview Schedule for *DSM-IV-TR Lifetime version (ADIS-IV-L*) [17]*.* Only patients who met clinical diagnostic criteria for a principal diagnosis of current generalized SAD (defined as greater than moderate anxiety/fear for five or more distinct social situations) or HC with no current or past history of DSM-IV disorders were eligible for participation.

**Table 1:** Demographic and clinical variables

	**SAD**	**HC**	***t*-value**	**Partial eta**^**2**^
	***n*** **= 27**	***n*** **= 27**		
Women (*n*)	12	13		
Age (mean years ± SD)	31.1 ± 7.6	32.6 ± 9.5	0.6	
Education (mean years ± SD)	16.3 ± 2.3	17.5 ± 1.5	2.0	
Ethnicity				
- Caucasian	12	17		
- Asian	5	8		
- Latino	6	2		
- Native American	1	0		
- Native Hawaiian	1	0		
- Filipino	1	0		
- African American	1	0		
LSAS-SR (Mean ± SD)	99.3 ± 11.8	15.3 ± 9.1	29.2^1^	0.94

^1^*P* <0.0001.

LSAS-SR = Liebowitz Social Anxiety Scale - Self-Report.

Patients and HC did not differ significantly in age or years of education (see Table 1). All participants were right-handed as assessed by the Edinburgh Handedness Inventory [18]. Potential patients were excluded if they reported current pharmacotherapy or psychotherapy, history of neurological disorders, and current psychiatric disorders (other than SAD, generalized anxiety disorder, agoraphobia without a history of panic attacks, dysthymia, or specific phobia). HC were not permitted to meet criteria for any current or past psychiatric disorders.

Among patients, current Axis-I co-morbidity included two with panic attacks, two with generalized anxiety disorder, two with dysthymia, and two with specific phobia. Past Axis-I co-morbidity included six with major depression, one with post-traumatic stress disorder, four with substance abuse, and one with eating disorder. Thirteen patients reported past (that is, ended >1 year ago) non-cognitive-behavioral psychotherapy, and seven reported past pharmacotherapy. The study was approved by the Stanford Medical Research Institutional Review Board (Protocol ID #79403). All participants provided informed consent in accordance with Stanford University Human Subjects Committee rules.

### Clinical assessment

To assess social anxiety symptom severity, participants completed the Liebowitz Social Anxiety Scale-Self-Report (LSAS-SR) [19]. This questionnaire assesses both fear and behavioral avoidance of social situations, and is widely used in the research of SAD [20].

To provide greater sensitivity, from 67 patients who were eligible for the study, we selected a subgroup of 27 SAD patients with the highest social anxiety symptom severity (range of LSAS-SR scores for the whole group: 66–102; and for the subgroup: 85–102). We compared this SAD subgroup to a group of 27 healthy controls.

### Experimental tasks

The three fMRI tasks have been described previously by Ziv et al. [10]. The tasks were composed of ‘React’ and ‘Reappraise’ conditions. Prior to scanning, participants were trained in how to react and to reappraise with stimuli not used in the MR scanner tasks. The instructions for the ‘React’ condition were to react normally without any attempt to control, modify, or regulate any reactions. During the ‘Reappraise’ condition, participants were instructed to try and down regulate negative emotion reactions by actively reinterpreting the meaning of the emotion inducing stimulus. We used reappraisal methods developed by Ochsner et al. [21].

After each trial, participants provided a negative emotion rating using a button response pad positioned in the participant’s right hand inside the magnet by responding to ‘How negative do you feel?’ (1 = not at all to 5 = very much). All tasks were programmed in Eprime (Psychology Software Tools, Inc.).

#### Faces task

**Figure 1:**
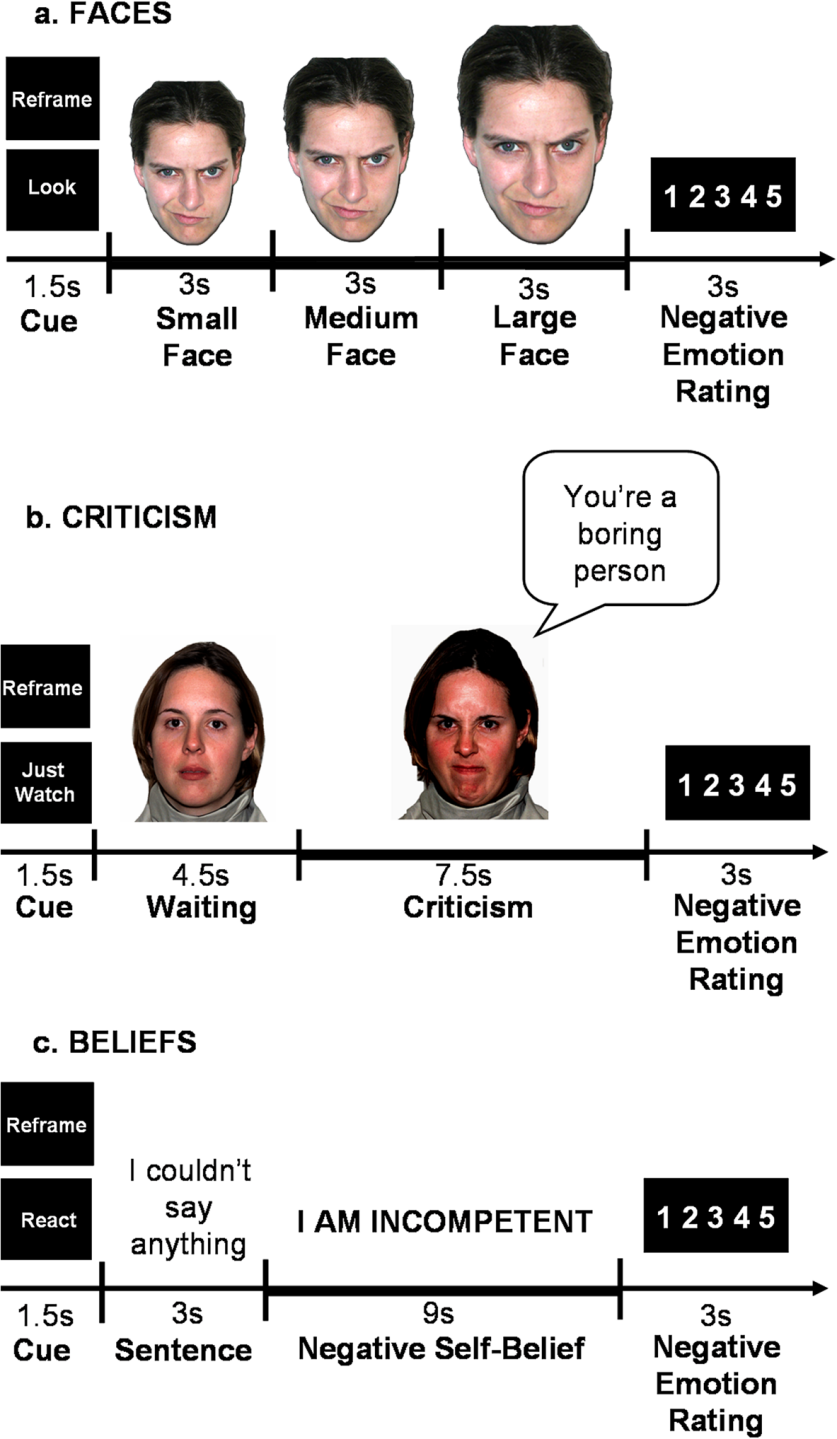
**The three socio-emotional tasks. (a)** Looming harsh faces (Faces); **(b)** Social criticism (Criticism); **(c)** Negative self-beliefs (Beliefs). Each task was composed of ‘React’ and ‘Reappraise’ trials, which consisted of: (1) a 1.5-s ‘Cue’; (2) a socio-emotional stimulus (a looming harsh face/ a video-clip of an actor delivering social criticism/ an autobiographical sentence + negative self-beliefs); and (3) a negative emotion rating scale. Participants were instructed to either react normally to the stimuli without any attempt to control, modify, or regulate their reactions (‘React’), or to try and down regulate negative emotion reactions by actively reinterpreting the meaning of the emotion inducing stimulus (‘Reappraise’).

This task consisted of 24 trials during which the participants viewed color photos of faces displaying Ekman facial action coded anger and contempt facial expressions [22]. Each trial consisted of a cue (Look or Reframe) lasting 1.5 s, a single harsh facial stimulus presented in color and appearing to move closer to the participant to simulate looming (every 3 s over a total of 9 s), and a negative emotion rating after the face stimulus terminated (3 s) (Figure 1a). The length of the entire task was 516 TRs, which is 12 min and 54 s (774 s). Participants were trained prior to the baseline scan to either react to the faces by engaging in the picture (‘Just let yourself feel’) and thinking: ‘This person is upset with me; angry with me’, or to reappraise their emotion, for example by thinking: ‘Maybe this person just had a bad day’.

#### Criticism task

The task consisted of videotaped actors delivering social criticism and social praise and harsh or happy evaluation-congruent facial expressions (Figure 1b). The stimuli were delivered by five male and five female actors (seven Anglo-Americans and three Asian-Americans), with an age range of 23 to 50 years. In each trial the participants were asked to either ‘Just Watch’ or ‘Reframe’ their reaction (1.5 s) during the social evaluation video clip (12 s). Each video clip had a 4.5-s waiting period during which the actor silently maintained a neutral facial expression followed by a 7.5-s evaluation period in which the actor delivered a social criticism or praise statement while displaying a harsh or positive facial expression. For the current study, only the 7.5-s evaluation period of the social criticism trials were included in the analysis. After each video clip, participants were cued to rate their current negative emotion (3 s). Each condition consisted of 16 trials delivered across two runs of 342 TRs, 8 min 35 s each (513 s). Participants were trained on the instructions prior to scanning. Participants were told to react to the social criticism by reflecting on how the statement represents something true about themselves, or to reappraise their emotion, for example by thinking: ‘this is not always true’, or ‘this is only a thought, not a fact’.

#### Beliefs task

This task consisted of five situations. The first was an experimenter-composed neutral situation that was used to obtain baseline emotion ratings for reading neutral statements. The neutral situation was followed by four participant-generated autobiographical social anxiety situations that were characterized by social anxiety, humiliation, and/or embarrassment. Prior to scanning session, participants composed a single paragraph describing the events, thoughts, and feelings for each situation, and provided situation-specific negative self-beliefs (NSBs). At the scanner, the participants were asked to either React to the negative self-beliefs, or to Reframe their reaction.

Three situations were presented in a first run lasting 374 TRs, 9 min 21 s (561 s), followed by two situations in a second run of 256 TRs, 6 min 24 s (534 s). Each situation consisted of an instruction to react/reframe (1.5 s), 16 sentences (3 s each,) in white font against a black background describing the situation, 10 NSBs (9 s each) embedded in the unfolding story in uppercase letters that flashed nine times (850 ms on + 150 ms off), and a negative emotion rating after each NSB (3 s) (Figure 1c). Participants were trained prior to scanning to react to the NSBs by reflecting on how the NSB represents something that is true about themselves, or to reappraise their reaction by thinking of a positive coping statement that directly challenges the thoughts (‘try to re-interpret the statement so it is less negative and toxic for you’).

### Image acquisition

Information about the fMRI image acquisition and data preprocessing have been described previously by Ziv et al. [8]. We used a General Electric 3-T Signa magnet with a T2*-weighted gradient echo spiral-in/out pulse sequence [23] and a custom-built quadrature ‘dome’ elliptical bird cage head-coil (GE Healthcare, Milwaukee, WI, USA). Head movements were minimized using a bite-bar and foam padding. Functional volumes (516 for faces, 684 for criticism, 630 for belief tasks) were obtained from 22 sequential axial slices (repetition time = 1500 ms, echo time = 30 ms, flip angle = 60°, field of view = 22 cm, matrix = 64 × 64, single-shot, resolution = 3.438 mm^2^ × 4.5 mm). Three-dimensional high-resolution anatomical scans were acquired using a fast spin-echo spoiled gradient recall (resolution = 0.8594 mm^2^ × 1.5 mm; field of view = 22 cm, frequency encoding = 256).

### FMRI data preprocessing

We used Analysis of Functional Neuroimages (AFNI) software [24] for preprocessing and statistical analysis. Preprocessing included an analysis of potential outliers, volume registration to a base image, motion correction, 4 mm^3^ isotropic Gaussian spatial smoothing, high-pass filtering (0.011 Hz), linear detrending, and conversion into BOLD signal percentage change in each voxel. In addition, the first four images of each functional run were excluded, to allow for T2* equilibration effects. For the Criticism and Belief tasks, the two functional runs were concatenated prior to statistical analysis. No volumes demonstrated motion in the x, y, or z directions in excess of ±1 mm. There was no evidence of stimulus-correlated motion, as assessed by correlations between condition-specific reference functions and x, y, z motion correction parameters.

### fMRI statistical analysis

Multiple-regression implemented with AFNI 3dDeconvolve included baseline parameters to remove mean, linear, and quadratic trends, and motion-related variance in the BOLD signal. Regressors for the React and Reappraise conditions were convolved with the Cohen’s gamma variate model of the hemodynamic response function [25]. Functional MRI BOLD signal intensity was computed as percentage of signal change, an effect size measure [(MR signal per voxel per time point/mean MR signal in that voxel for the entire functional run) × 100].

Individual brain maps were converted to Talairach atlas space [26] and second-level group statistical parametric maps were produced according to a random-effects model. To correct for multiple comparisons, AlphaSim, a Monte Carlo simulation bootstrapping program in the AFNI library, was used to protect against false positives [27]. This method uses a voxel-wise and cluster volume joint-probability threshold to establish a cluster-wise false-positive cluster detection level. The cluster statistical threshold for the between-group analyses consisted of a voxel-wise *P* <0.005 and cluster volume >244 mm^3^ (6 voxels × 3.438 mm^3^) to protect against false-positive cluster detection at *P* <0.01.

To examine differential responses in BOLD signal magnitude, we conducted a whole-brain 2 Group (SAD, HC) between-group t-test of Reappraise *versus* React in SAD *versus* HC. To identify common responses, we ran a one-sample t-test of Reappraise *versus* React in SAD, and separately in HC.

To further examine the between-group differences in the BOLD responses during reappraisal, we ran between-group t-tests for early (first three time points, 0 to 4.5 s) and late (last two/three time points, 4.5 to 7.5 s or 4.5 to 9 s) BOLD responses for the contrast of Reappraise *versus* React. This analysis was conducted in each of the brain regions showing a between-group difference, and had several sub-steps: (1) creating masks from each of the clusters surviving the threshold of the between-group t-test, separately for each task; (2) extracting the percent signal change from each of these regions, at the individual subject level, for the Reappraise condition and for the React condition; (3) calculating the percent signal change for Reappraise minus React; (4) averaging (for each subject) the percent signal change for the first three time points, and for the last three time points; and (5) running between-group t-tests (SAD *versus* HC) separately for the early and late averaged responses, for each task.

In a secondary analysis, we examined brain regions showing BOLD responses common to SAD and HC across the three tasks, and ran between-group t-tests on the contrast of Reappraise *versus* React, for early and late responses, separately, for each task.

## Results

### Faces task

#### Behavioral responses: negative emotion ratings

A between-group t-test revealed no significant differences (*P* >0.23) in percent reduction in negative emotion between HC (30 %) and SAD (23 %) when reappraising faces (Additional file 1: Figure S1a).

#### Brain responses: BOLD signal magnitude

##### Differential responses

**Figure 2:**
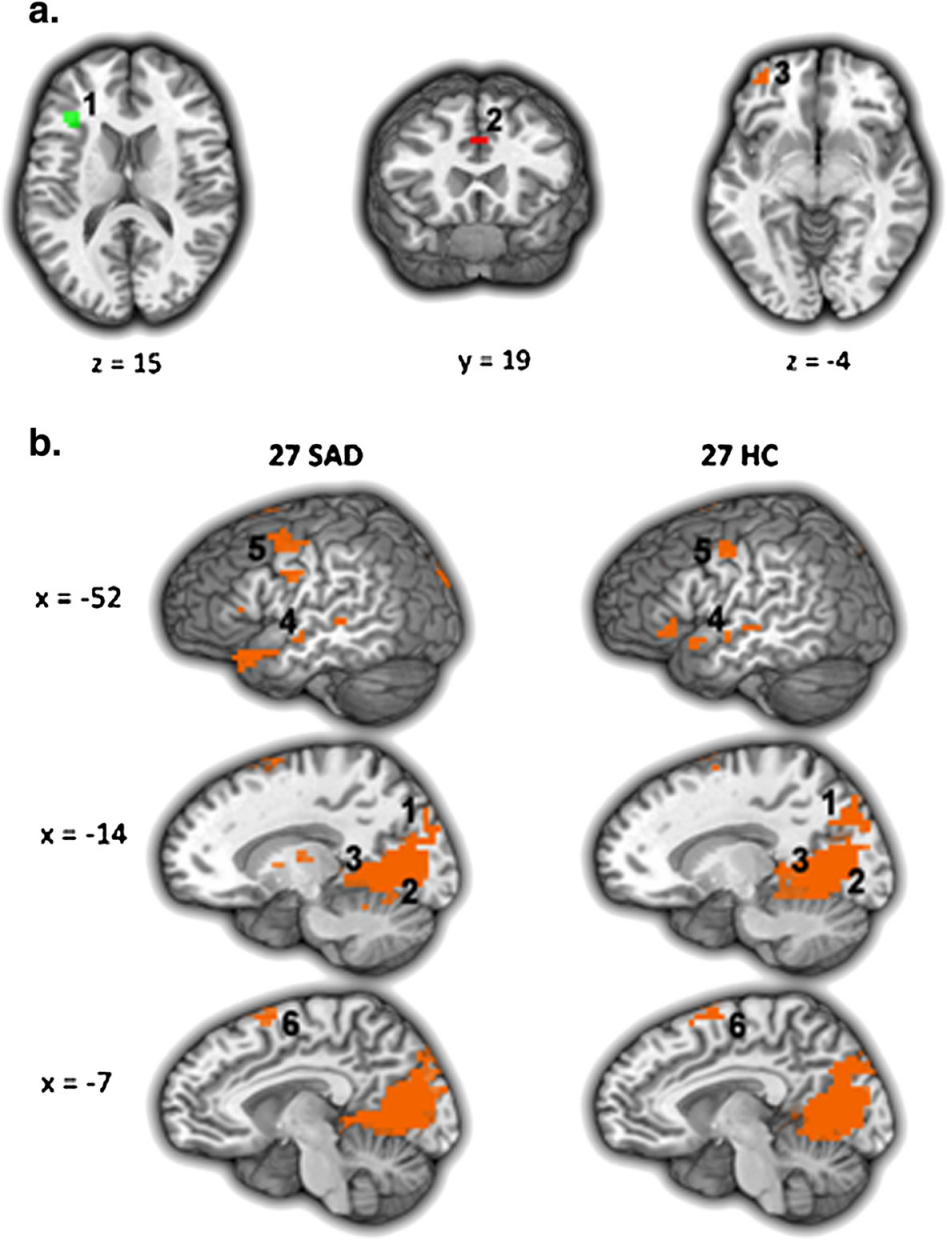
**Faces task: brain responses during Reappraise *versus *React in SAD and HC. (a)** Differential BOLD responses in patients with SAD *versus* healthy controls (HC > SAD). 1 = left inferior frontal gyrus, 2 = dorsal ACC, 3 = left lateral orbitofrontal cortex. **(b)** Common BOLD responses in patients with SAD and healthy controls. 1 = left cuneus, 2 = left lingual gyrus, 3 = left parahippocampus, 4 = left middle temporal gyrus, 5 = left precentral gyrus, 6 = dorsomedial PFC. Statistical threshold for BOLD responses: t-value threshold ≥2.93, voxel *P* <0.005, minimum cluster volume threshold ≥244 mm^3^ (6 voxels × 3.438 mm^3^), cluster-wise *P* <0.01.

Compared to HC, patients had lesser reappraisal-related BOLD responses in the left inferior frontal gyrus (IFG), dorsal anterior cingulate cortex (dACC), and left lateral orbitofrontal cortex (LOFC) (Figure 2a).

##### Common responses

Separate one-sample t-tests of Reappraise *versus* React demonstrated increased BOLD responses in both groups in bilateral cuneus, precuneus, lingual gyrus, and hippocampus, and in left middle temporal gyrus, left precentral gyrus, and dorsomedial PFC (Figure 2b). See Table 2 for both differential and common responses.

**Table 2:** **Faces task: differential and common BOLD responses for Reappraise *versus *** **React**

	**x y z**	**Vol (mm**^**3**^**)**	***t*-value**
**Between-group differential responses**			
**SAD > HC: Reappraise > React-none**			
**HC > SAD: Reappraise > React**			
Left inferior frontal gyrus	-34, 25, 15	426	3.56
Dorsal anterior cingulate gyrus	0, 18, 33	372	3.46
Left lateral orbitofrontal cortex	-31, 52, -6	372	3.63
**Within-group responses**^**1**^			
**SAD only: Reappraise > React**			
*Right cuneus*	3, -82, 39	57,444	3.39
*(Left cuneus, bilateral precuneus, bilateral lingual gyrus, bilateral parahippocampal gyrus)*			
Left superior temporal gyrus BA38	-48, 28, -19	3,191	3.30
Left Superior Temporal Gyrus BA21	-65, -20, -2	1,330	3.76
*Left dorsomedial PFC*	-3, 11, 63	1,330	3.38
*Left precentral gyrus BA6*	-52, -3, 29	1,170	3.61
Right middle temporal gyrus BA21	55, -3, -9	1,117	3.64
Left putamen	-21, 4, 5	1,117	4.26
Right superior temporal gyrus BA38	45, 21, -16	1,011	3.56
Left inferior frontal gyrus BA45	-55, 31, 8	532	3.09
Left superior temporal gyrus	-65, -13, -2	479	3.72
Left thalamus	-14, -13, -8	426	4.05
Right thalamus	14, -13, 12	426	4.15
Right superior temporal gyrus	38, 7, -12	372	3.12
*Left middle temporal gyrus*	-55, -10, -9	372	3.75
**HC only: Reappraise > React**			
*Left lingual gyrus*	-3, -58, 5	67,125	7.08
*(Right lingual gyrus, bilateral cuneus, bilateral precuneus, bilateral parahippocampus)*			
*Left dorsomedial PFC*	-3, 4, 63	3,191	3.73
*Left precentral gyrus BA4/6*	-55, -3, 50	1,808	3.21
Left supramarginal gyrus BA40	-65, -48, 19	1,011	3.83
Left superior temporal gyrus	-55, -17, -2	798	5.05
Left inferior frontal gyrus	-52, 25, -6	745	3.62
Left superior temporal gyrus BA22	-62, -34, 12	745	3.10
Right superior temporal gyrus BA38	41, -24, 1	691	3.53
Left superior temporal gyrus BA38	-52, 14, -9	585	3.40
Right precentral gyrus BA6	62, 4, 19	426	3.69
*Left middle temporal gyrus*	-55, -6, -6	426	4.69

^1^Regions showing increased BOLD response during Reappraise *versus* React in both SAD and HC (common responses) are marked in italics.

Note. *t*-value threshold ≥2.932, voxel *P* <0.005, minimum cluster volume threshold ≥244 mm^3^ (6 voxels × 3.438 mm^3^), cluster *P* <0.01.

BA = Brodmann area, xyz = Talairach and Tournoux coordinates of maximum BOLD signal intensity voxel.

#### Brain responses: BOLD signal temporal dynamics

**Figure 3:**
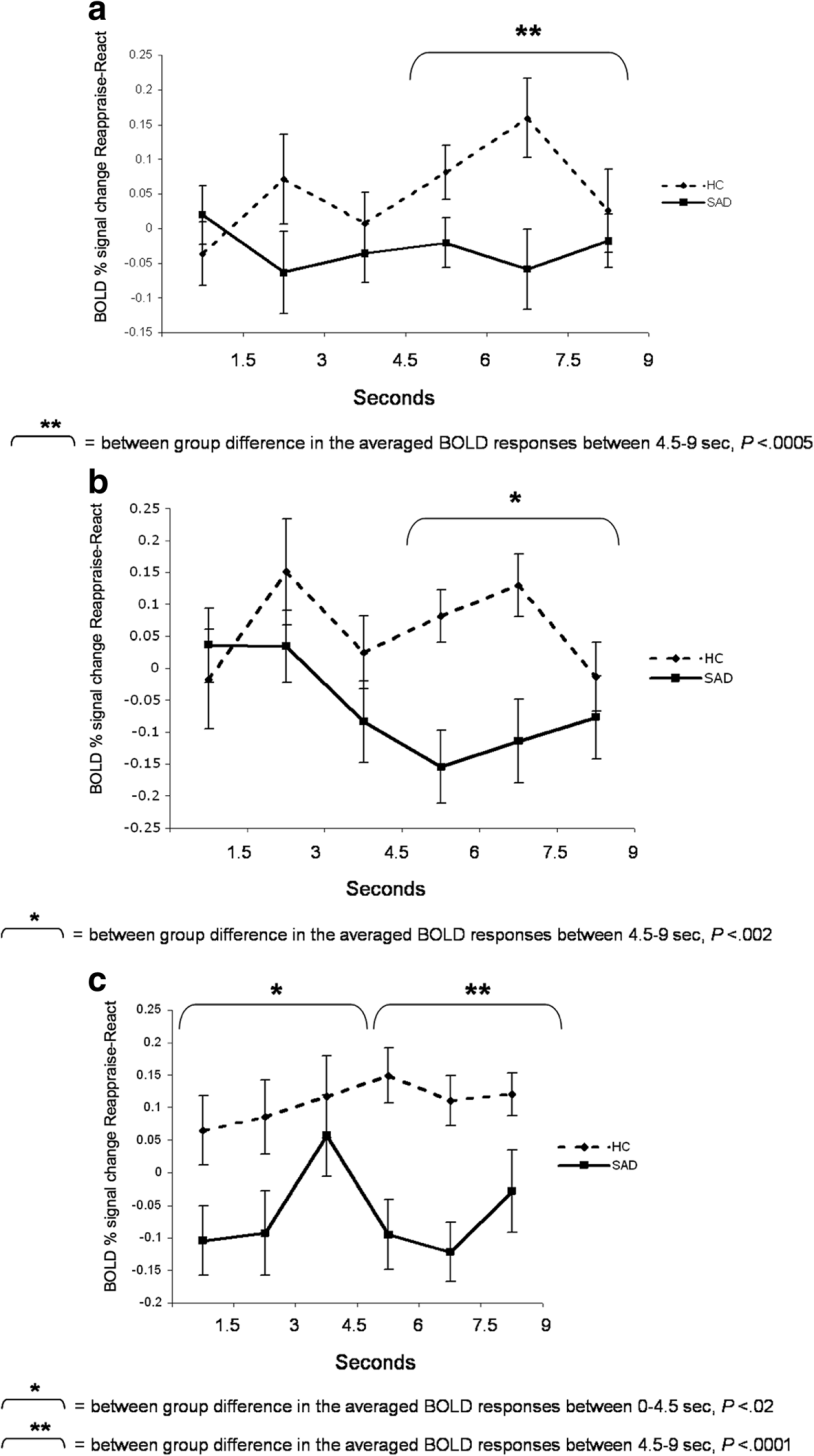
**Faces task: temporal dynamics of the BOLD response during Reappraise *versus *React in SAD and HC.** Asterisks represent a significant between-group difference in the average of the first three time points (early) or last three time points (late) BOLD responses. **(a)** Left inferior frontal gyrus; **(b)** Dorsal ACC; **(c)** Left lateral orbitofrontal cortex.

To examine between-group differences in the BOLD responses during reappraisal, we conducted between-group t-tests for early (first three time points; 0–4.5 s) and late (last three time points; 4.5-9 s) BOLD responses for the Reappraise *versus* React contrast in the left IFG, dACC, and left LOFC. Between-group t-tests revealed a significantly greater late responses in HC, compared to SAD, in the left IFG (mean HC = 0.09 *vs.* mean SAD = -0.03, t_52_ = -3.70, *P* <0.0005) and the dACC (mean HC = 0.07 *vs*. mean SAD = -0.11, t_52_ = -3.26, *P* <0.002), with no between-group differences in early responses (Figures 3a, b). For the left LOFC, a between-group t-test showed that, compared to SAD, HC had greater early (mean HC = 0.09 *vs*. mean SAD = -0.05, t_50_ = -2.34, *P* <0.02) and late (mean HC = 0.13 *vs*. mean SAD = -0.09, t_50_ = -5.10, *P* <0.0001) responses for the Reappraisal *versus* React contrasts (Figure 3c).

### Criticism task

#### Behavioral responses: negative emotion ratings

A between-group t-test revealed a trend towards greater percent reduction in negative emotion ratings for HC (23 %, SD = 18) than SAD (14 %, SD = 17; t_53_ = -1.93, *P* <0.059) when reappraising criticism (Additional file 1: Figure S1b).

#### Brain responses: BOLD signal magnitude

##### Differential responses

**Figure 4:**
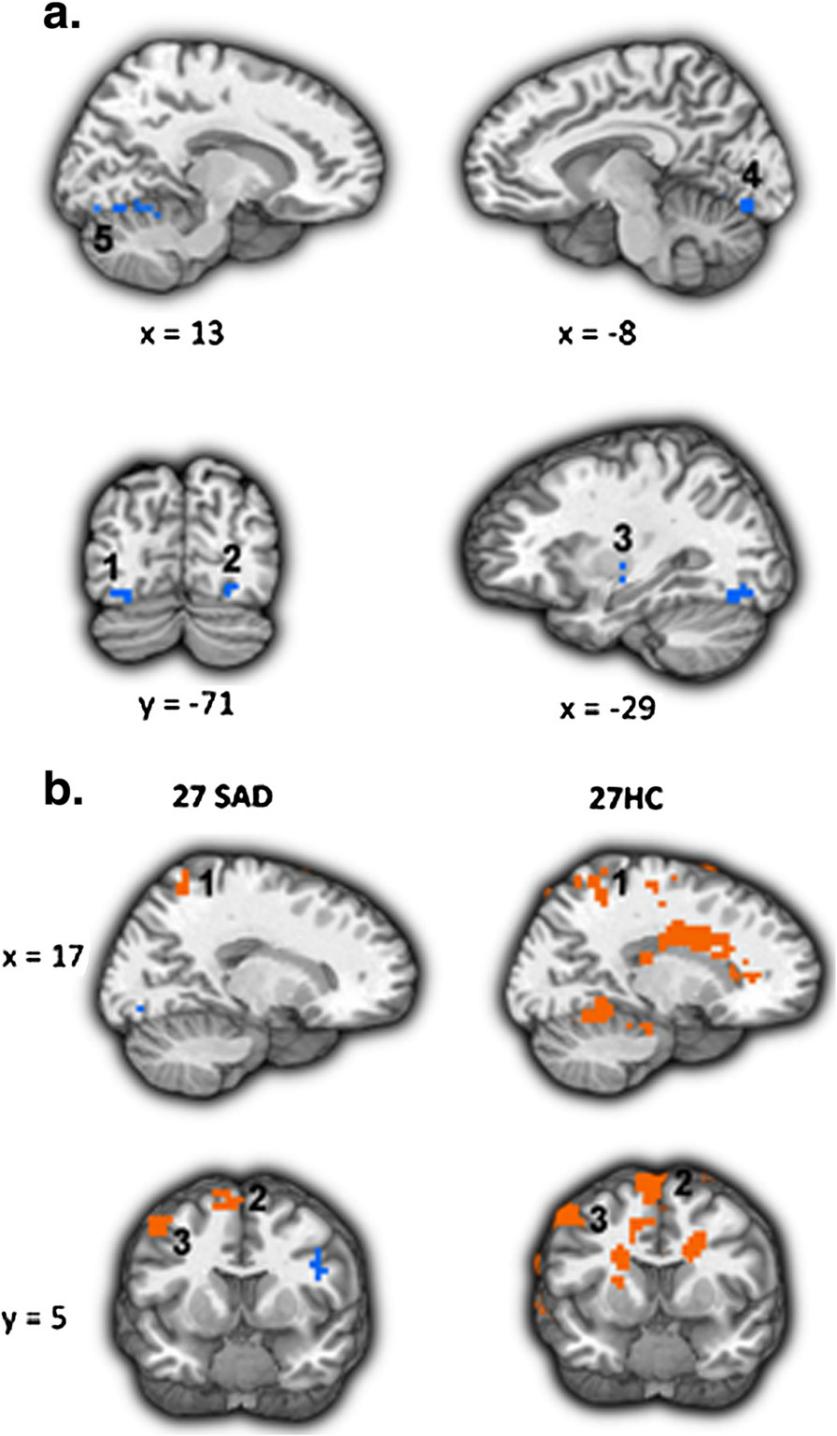
**Criticism task: brain responses during Reappraise *versus *React in SAD and HC. (a)** Differential BOLD responses in patients with SAD *versus* healthy controls (HC > SAD). 1 = left fusiform gyrus, 2 = right fusiform gyrus, 3 = left putamen, 4 = left lingual gyrus, 5 = right cerebellum. **(b)** Common BOLD responses in patients with SAD and healthy controls. 1 = right precuneus, 2 = dorsomedial PFC, 3 = left dorsolateral PFC. Statistical threshold for BOLD responses: t-value threshold ≥2.93, voxel *P* <0.005, minimum cluster volume threshold ≥244 mm^3^ (6 voxels × 3.438 mm^3^), cluster-wise *P* <0.01.

Compared to HC, patients had lesser reappraisal-related BOLD responses in bilateral fusiform gyrus, left lingual gyrus, left putamen, and right cerebellum (Figure 4a).

##### Common responses

Separate one-sample t-tests of Reappraise *versus* React demonstrated increased BOLD responses in both groups in the right precuneus, dorsomedial PFC, and left dorsolateral PFC (Figure 4b). See Table 3 for both differential and common responses.

**Table 3:** **Criticism task: differential and common BOLD responses for Reappraise *versus *** **React**

	**x y z**	**Vol (mm**^**3**^**)**	**t-value**
**Between-group differential responses**			
**SAD > HC: Reappraise > React - none**			
**HC > SAD: Reappraise > React**			
Left fusiform gyrus	-24, -68, -12	904	3.16
Right cerebellum	3, -68, -9	745	3.01
Right fusiform gyrus	24, -75, -12	585	4.13
Left putamen	-28, -13, -6	479	2.96
Left lingual gyrus	-7, -75, -12	426	3.28
Right cerebellum	24, -51, -16	372	2.94
Right cerebellum	17, -58, -12	372	3.04
**Within-group responses**^**1**^			
**SAD only: Reappraise > React**			
*Left dorsomedial PFC*	-3, 11, 60	2,500	4.27
*Left dorsolateral PFC*	-48, 7, 50	1,170	3.59
*Right precuneus*	17, -55, 63	745	3.36
Right parahippocampal gyrus	24, -44, -6	372	3.25
**HC only: Reappraise > React**			
Left lingual gyrus	-3, -58, 5	13,936	3.21
(Right lingual gyrus, bilateral fusiform gyrus, bilateral cerebellum)			
Right precentral gyrus BA6/BA4	28, -17, 63	9,308	3.14
*Left dorsomedial PFC*	-3, 11, 67	6,915	3.34
*Left dorsolateral PFC*	-52, 4, 50	3,776	3.83
Left supramarginal gyrus	-62, -58, 15	3,351	4.09
Left middle frontal gyrus	-45, 21, 29	2,234	3.42
Right superior parietal cortex/precuneus	24, -55, 60	2,128	3.31
Left superior parietal cortex/precuneus	-24, -75, 53	1,808	4.18
Left inferior frontal gyrus BA45	-55, 25, -2	1,755	3.25
Left medial frontal gyrus	-3, 7, 43	1,170	3.15
*Right precuneus*	28, -72, 22	1,064	3.45
Left superior temporal gyrus	-65, -174, -2	1,064	3.79
Left middle frontal gyrus	-31, 49, 12	851	3.14
Left precuneus	-31, -89, 19	745	3.34
Left anterior insula	-48, 11, 5	745	3.53
Left precentral gyrus BA4	-24, -24, 56	691	3.56
Left thalamus	-3, -3, 12	638	3.34
Left angular gyrus	-48, -61, 36	585	3.12
Left superior temporal gyrus	-38, -55, 29	319	3.38
Left putamen	-31, -10, -2	319	3.28
Left middle temporal gyrus	-52, -34, 5	319	3.33

^1^Regions showing increased BOLD response during Reappraise *versus* React in both SAD and HC (common responses) are marked in italics.

Note. *t*-value threshold ≥2.93, voxel *P* <0.005, minimum cluster volume threshold ≥244 mm^3^ (6 voxels × 3.438 mm^3^), cluster *P* <0.01.

BA = Brodmann area, xyz = Talairach and Tournoux coordinates of maximum BOLD signal intensity voxel.

#### Brain responses: temporal dynamics

To examine the between-group differences in the BOLD signal timing during reappraisal, we conducted between-group t-tests for early (first three time points; 0–4.5 s) and late (last two time points; 4.5-7.5 s) BOLD responses for the Reappraise *versus* React conditions in left and right fusiform gyrus, left lingual gyrus, left putamen, and right cerebellum.

**Figure 5:**
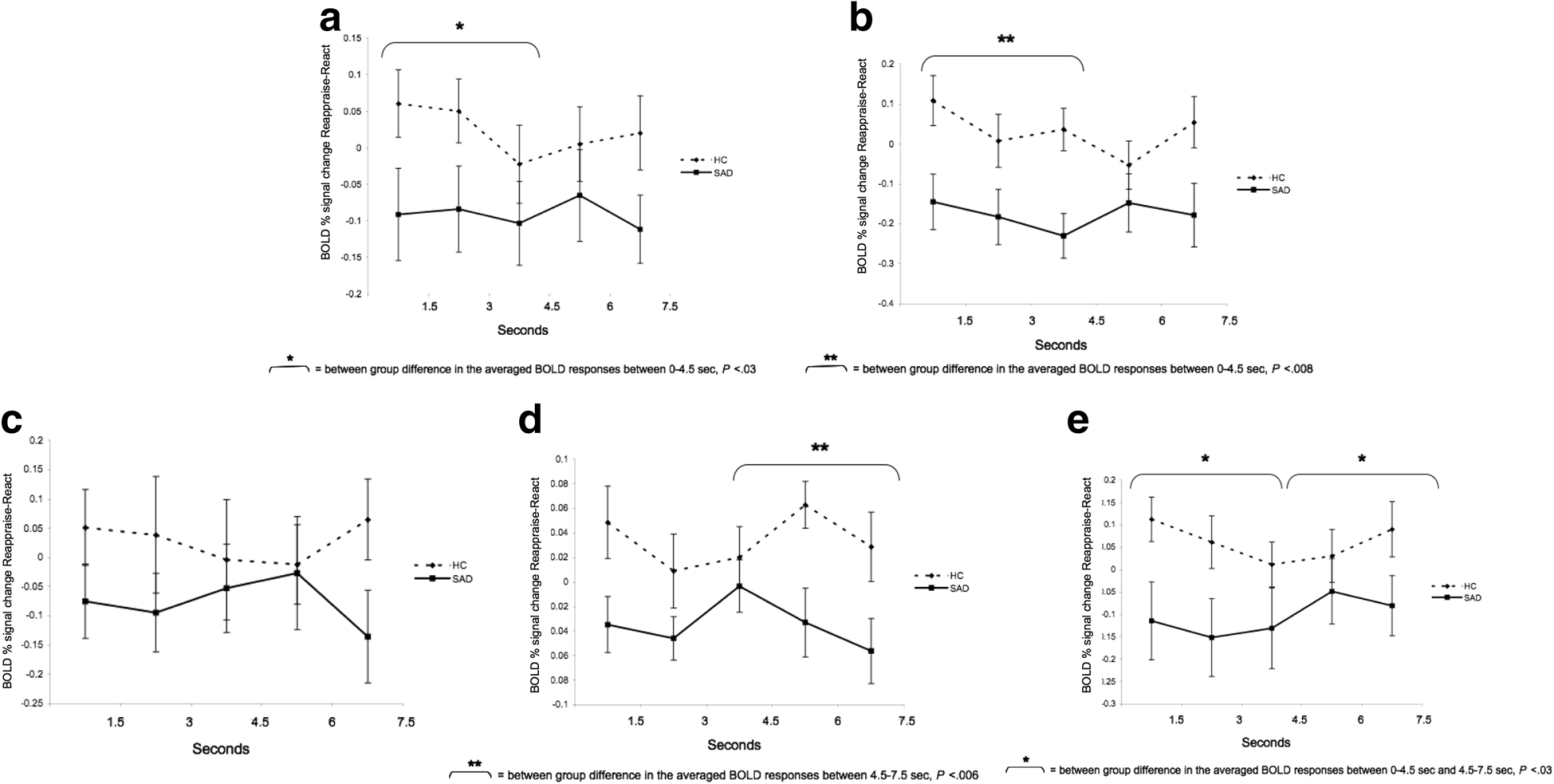
**Criticism task: temporal dynamics of the BOLD response during Reappraise *versus *React in SAD and HC.** Asterisks represent a significant between-group difference in the average of the first three time points (early) or last two time points (late) BOLD responses. **(a)** Left fusiform gyrus; **(b)** Right fusiform gyrus; **(c)** Left lingual gyrus; **(d)** Left putamen; **(e)** Right cerebellum.

Between-group t-tests revealed a significant greater early response in HC, compared to SAD, in the left (mean HC = 0.03 *vs*. mean SAD = -0.09; t_48_ = -2.20, *P* <0.03) and right (mean HC = 0.05 *vs*. mean SAD = -0.19, t_48_ = -2.78, *P* <0.008) fusiform gyrus. For the left lingual gyrus, there were no early or late significant between-group differences (All *Ps* > 0.26). Significant increased late response in HC, compared to SAD, was found in the left putamen (mean HC = 0.05, mean SAD = -0.05, t_50_ = -2.88, *P* <0.006). Time course analyses for the right cerebellum showed both early (mean HC = 0.05 *vs*. mean SAD = -0.15, t_49_ = -2.32, *P* <0.03) and late (mean HC = 0.05 *vs*. mean SAD = -0.15, t_49_ = -2.22, *P* <0.03) increased responses in HC, compared to SAD, during reappraisal (Figure 5a-e)^a^.

### Beliefs task

#### Behavioral responses: negative emotion ratings

A between-group t-test revealed greater percent reduction in negative emotion ratings for HC (31%, SD = 16.2) than SAD (19%, SD = 19; t49 = -2.52, *P* <0.02) when reappraising beliefs (Additional file 1: Figure S1c).

#### Brain responses: BOLD signal magnitude

##### Differential responses

**Figure 6:**
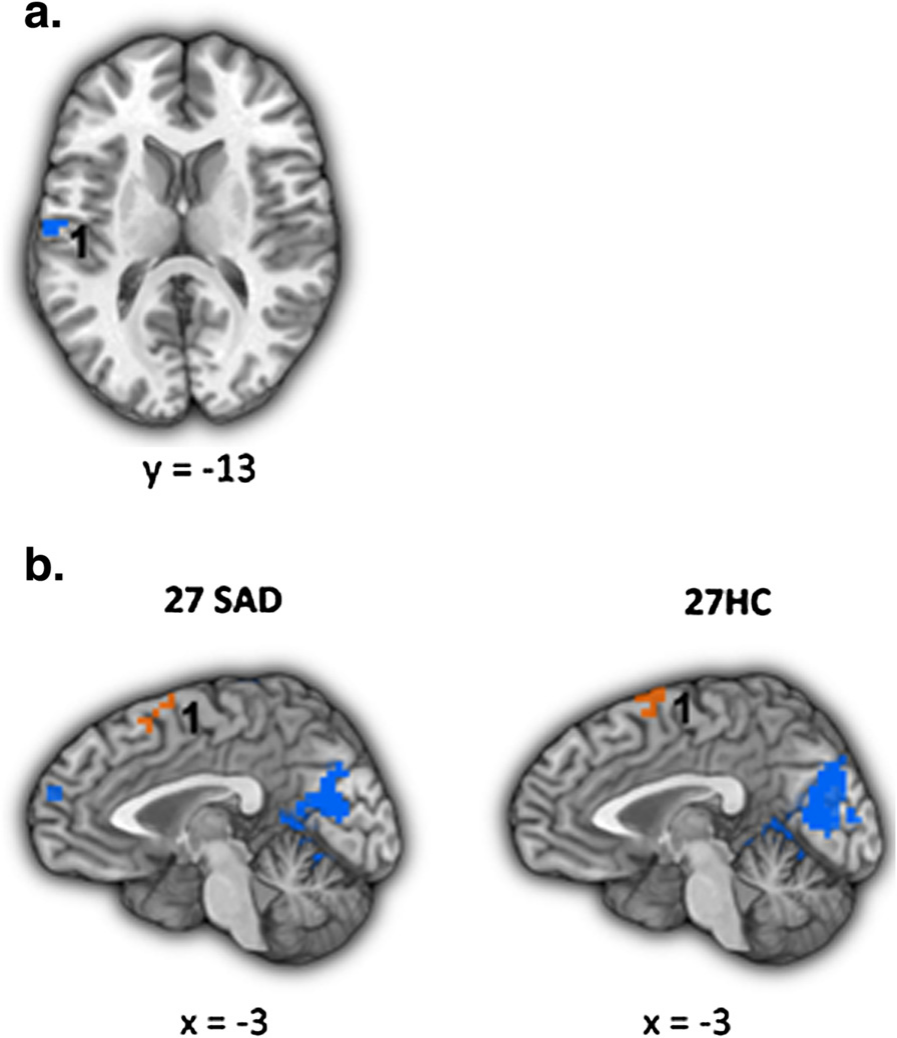
**Beliefs task: brain responses during Reappraise *versus *React in SAD and HC. (a)** Differential responses in patients with SAD *versus* healthy controls (HC > SAD). 1 = left superior temporal gyrus. **(b)** Common BOLD responses in patients with SAD and healthy controls. 1 = dorsomedial PFC. Statistical threshold for BOLD responses: t-value threshold ≥2.93, voxel *P* <0.005, minimum cluster volume threshold ≥244 mm^3^ (6 voxels × 3.438 mm^3^), cluster-wise *P* <0.01.

Compared to HC, patients had lesser BOLD responses in the left superior temporal gyrus (STG) (Figure 6a).

##### Common responses

Separate one-sample t-tests of Reappraise *versus* React demonstrated increased response in both groups in the dorsomedial PFC (Figure 6b). See Table 4 for both differential and common responses.

**Table 4:** **Beliefs task: differential and common BOLD responses for Reappraise *versus *** **React**

	**x y z**	**Vol (mm**^**3**^**)**	***t*-value**
**Between-group differential responses**			
**SAD > HC: Reappraise > React - none**			
**HC > SAD: Reappraise > React**			
Left superior temporal gyrus BA41	-58, -13, 12	585	2.96
**Within-group responses**^**1**^			
**SAD only: Reappraise > React**			
*Left dorsomedial PFC*	-3, 7, 60	1,596	3.16
Left inferior frontal gyrus BA45	-55, 25, 8	638	3.81
Left dorsolateral PFC	-41, 7, 50	638	3.57
**HC only: Reappraise > React**			
*Left dorsomedial PFC*	-3, 11, 63	1,170	3.09

^1^Regions showing increased BOLD response during Reappraise versus React in both SAD and HC (common responses) are marked in italics.

Note. *t*-value threshold ≥2.93, voxel *P* <0.005, minimum cluster volume threshold ≥244 mm^3^ (6 voxels × 3.438 mm^3^), cluster *P* <0.01.

BA = Brodmann area, xyz = Talairach and Tournoux coordinates of maximum BOLD signal intensity voxel.

#### Brain responses: temporal dynamics

**Figure 7:**
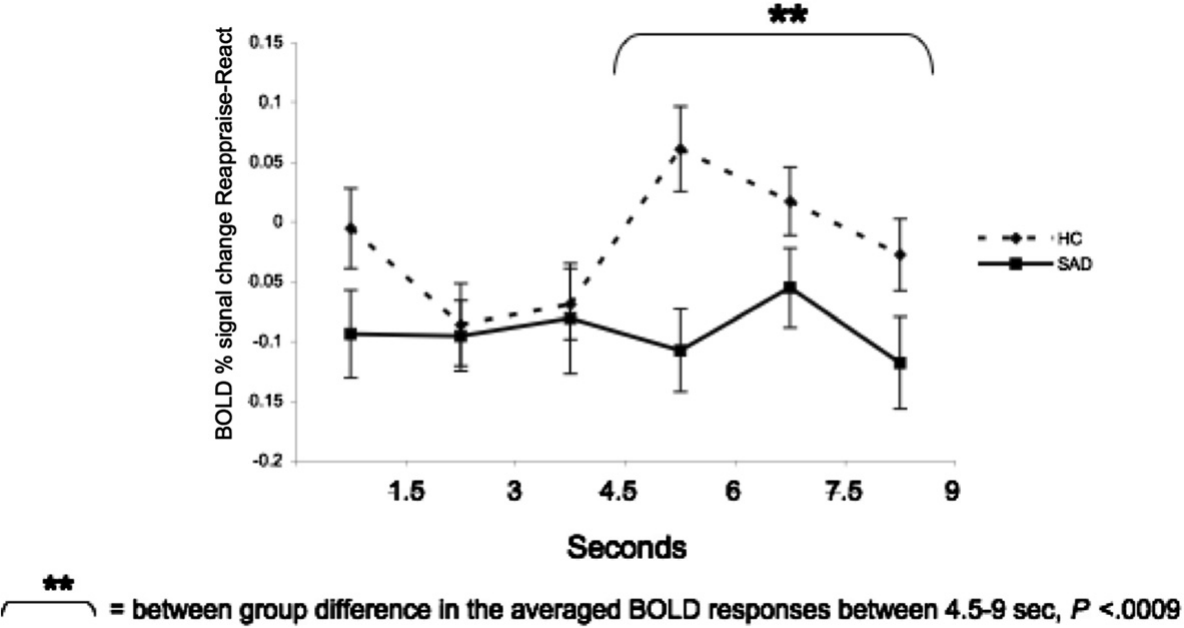
**Beliefs task: temporal dynamics of the left STG BOLD responses during Reappraise *versus *React in SAD and HC.** Asterisks represent a significant between-group difference in the average of the last three time points (late) BOLD responses.

To examine between-group differences in the BOLD responses during reappraisal of beliefs, we conducted between-group t-tests for early (first three time points; 0–4.5 s) and late (last three time points; 4.5-9 s) BOLD responses for the Reappraise *versus* React conditions in the left STG. This analysis revealed a significant increased late response in HC, compared to SAD (mean HC = 0.02 *vs*. mean SAD = -0.09, t_51_ = -3.52, *P* <0.0009) (Figure 7).

### Secondary analyses

**Figure 8:**
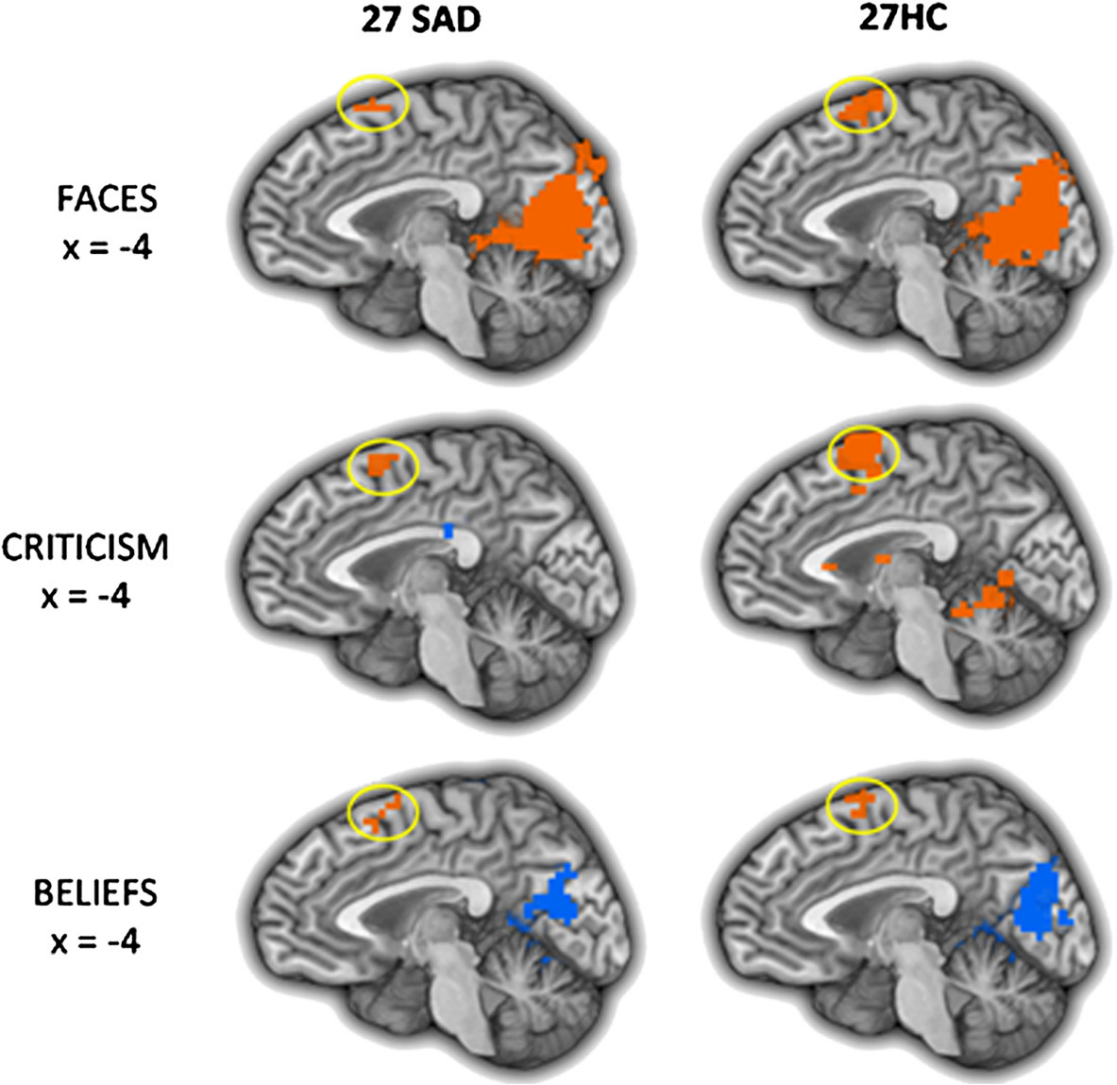
**Dorsomedial PFC BOLD responses during the three socio-emotional tasks.** The dorsomedial PFC (DMPFC) showed increased activity in both patients with SAD and HC, during reappraisal of faces, criticism, and beliefs.

Examination of the reappraisal-related brain responses common for SAD and HC revealed only one region, the dorsomedial PFC (DMPFC), which showed reappraisal-related increased activity in both groups, in all three tasks (Figure 8). To examine the temporal dynamics of the BOLD response, and its potential association with between-group differences, we conducted two between-group t-tests: one for early and one for late BOLD responses during the Reappraise *versus* React conditions in this region, separately for each task.

**Figure 9:**
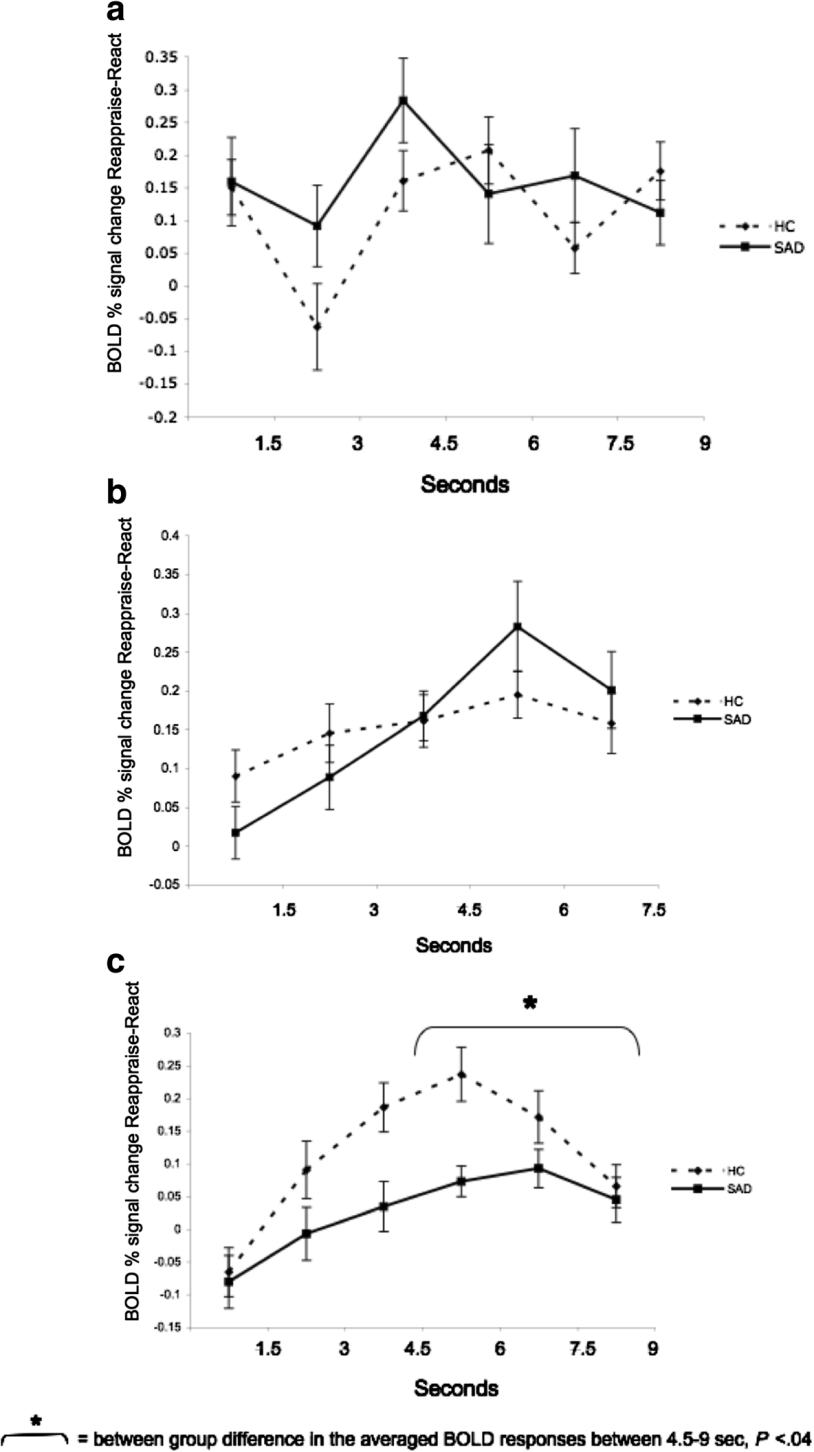
**Temporal dynamics of the dorsomedial PFC BOLD responses during Reappraise *versus *React Faces (a), Criticism (b), and Beliefs (c) in SAD and HC.** No significant between-group differences were found in early or late DMPFC activity when reappraising faces and criticism. A significant between-group difference was found in late DMPFC activity when reappraising beliefs (P <0.04).

There were no significant between-group differences in early or late responses for the contrast of Reappraise *versus* React Faces (*P*s >0.08) and Criticism (*P*s >0.26) (Figure 9a,b). However, for Beliefs, compared to HC, patients had lesser late BOLD responses (mean HC = 0.17, mean SAD = 0.07, t_50_ = -2.12, *P* <0.04) (Figure 9c).

**Figure 10:**
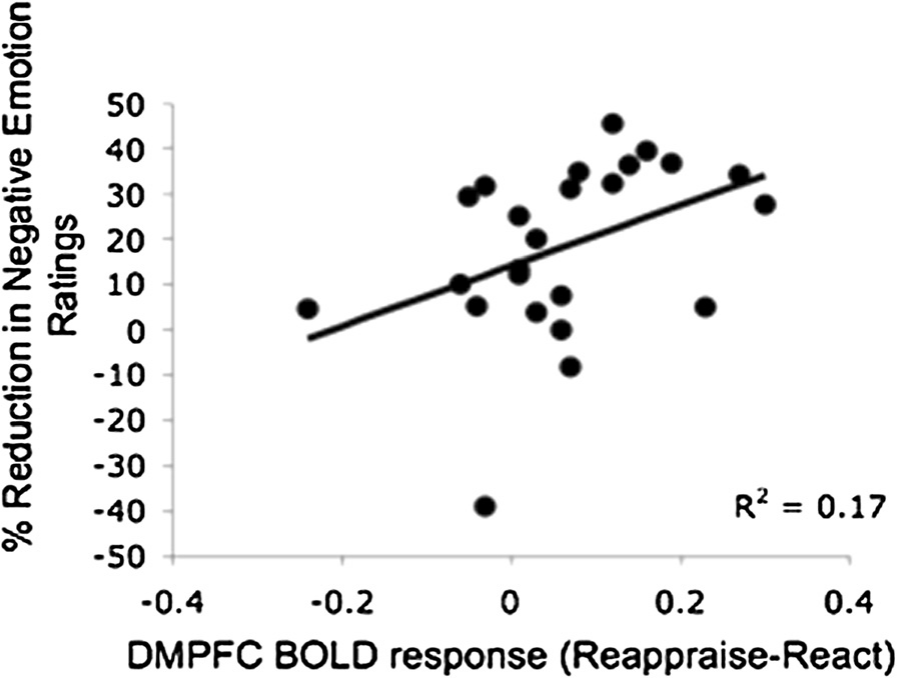
**Beliefs task: association between dorsomedial PFC BOLD responses during Reappraise *versus *React and percent reduction in negative emotion ratings.** When reappraising beliefs, DMPFC BOLD responses were positively correlated with % reduction in negative emotion ratings in SAD patients (r = 0.42, *P* <0.04).

Greater late DMPFC responses were associated with greater reduction in negative emotion ratings in SAD patients (r = 0.42, *P* <0.04), but not HC (r = -0.32, *P* = 0.13; Zdiff = 2.52, *P* <0.05) (Figure 10).

## Discussion

We tested the hypothesis that dysfunctional emotion regulation processes in SAD patients are associated with altered reappraisal-related activity in prefrontal brain regions. We focused on both BOLD signal magnitude and temporal dynamics during reappraisal of three different socio-emotional stimuli in patients with SAD *versus* healthy controls. Results suggest distinct behavioral and neural effects related to each of the socio-emotional tasks.

### Behavioral correlates of reappraisal in SAD

Behaviorally, the results of this study indicated a smaller percent reduction of negative emotion in patients with SAD compared to HC, but only during the reappraisal of idiographic social anxiety-related negative self-beliefs. Though it has long been thought that patients with SAD have problems with down regulating negative emotions [2,3,5,28], the behavioral findings in our study suggest a more nuanced picture of deficits in reappraisal. Specifically, the emotion regulation deficits were most pronounced when facing the negative self-beliefs. These idiographic stimuli, each linked to autobiographical social anxiety-related situations, are highly potent - in our previous report [10], using the same stimuli and the same sample, negative self-beliefs were associated with the greatest increase in negative emotion, compared to faces and criticism, in both SAD and HC. This suggests greater emotional effect for these stimuli, which might be related with the difficulty in down regulating its associated negative emotions.

Our finding is partially in line with the results of the one other study examining cognitive regulation of negative self-beliefs in SAD [3]. In that study, although no difference was found between patients and controls in the amount of reappraisal-related reduction of negative emotion, greater social anxiety symptom severity was associated with lesser down regulation of negative emotion in patients, suggesting that severity of social anxiety contributes to deficits in cognitive reappraisal of negative beliefs.

### Neural correlates of reappraisal in SAD

Neurally, based on the prior findings in patients with SAD [3,5] and non-clinical populations [21], we hypothesized that, relative to HC, patients would have reduced recruitment of PFC regions during reappraisal. The between-group analyses partially confirmed our hypothesis. While there was PFC activation during reappraisal in both patients and controls during all three tasks, between-group differential PFC activation was found only during the reappraisal of faces, with no differential PFC responses when reappraising beliefs and criticism.

#### Reappraisal of looming harsh faces

When reappraising faces, compared to HC, patients had reduced activity in a PFC network that included left IFG, dACC, and lateral OFC. In these regions, time course analyses revealed greater BOLD response during the second half of the trial in HC compared to patients. This increased late responses in HC when reappraising looming harsh faces might be related to integrated cognitive processes that rely on linguistic (left IFG), cognitive control/attention (dACC), and evaluation/response selection (lateral OFC) processes [29]. In patients, the reduced late recruitment of prefrontal regulatory brain regions suggests deficits in reappraisal processes, especially of faces displaying contempt, anger, or disapproval. The use of harsh faces as an emotional probe in patients with SAD has ecological validity given that SAD is characterized by fear of interpersonal situations and a tendency to avoid eye contact [30]. Cognitive behavioral models of social anxiety suggest that patients with SAD manifest increased attentional focus on others’ facial expressions and negative evaluation during social situations [31]. Supporting the models, behavioral studies have reported that patients with SAD tend to remember critical faces better than accepting ones [32], and scan faces with a different pattern of eye movements than is used by healthy controls [33]. The results of the current study accord with these models of social anxiety and extend the current theory by suggesting a delayed regulatory deficit in SAD.

#### Reappraisal of social criticism

During reappraisal of criticism, the left putamen exhibited increased reappraisal-related activity in HC, compared to SAD, during the second half of the trials. Previous studies implicated the putamen as involved in implicit learning, and specifically in prediction of future reward [34]. Recently, the left putamen was shown to be active when participants had to explicitly mirror observed emotional facial expressions [35]. The authors of this study suggested that the putamen is involved in the establishment of a successful social connection with another person. In the current study, it might be that HC, but not patients, were able to form a relatively more positive (or less negative) interpretation of the social feedback delivered by the actor following the reappraisal instruction. For patients with SAD, difficulties implementing cognitive reappraisal might make it harder for them to relate to the person in the video clip in a positive way, and prevent them from seeing the social situation as a potential rewarding event.

Interestingly, time course analyses of the fusiform gyrus, lingual gyrus, and cerebellum responses revealed that the between-group differences derived less from increased reappraisal-related responses in HC, and more from increased reactivity-related responses in patients with SAD. These results are in line with electrophysiological studies demonstrating early hyper-vigilance followed by attentional avoidance in adults with SAD when facing social threat stimuli [36]. Overall, this result confirms previous findings of increased activity in visual attention-related regions in SAD when reacting to social relevant stimuli [3].

#### Reappraisal of autobiographical negative self-beliefs

During reappraisal of beliefs, the between-group results indicated increased reappraisal-related activity in the left STG in healthy controls. The STG also manifested a late activity peak for HC, compared to SAD. The STG is related to social cognition, namely, the ability to attribute mental states to the self and others. During cognitive reappraisal, attending to one’s own emotional state or to those of others is crucial to be able to monitor the process of changing the affective state [37]. Thus, greater activity in this region in HC, compared to patients, might be associated with the patients’ reduced ability to regulate their cognitions when facing their self-created negative self-beliefs.

### Temporal dynamics of reappraisal

Across the three tasks, the results of the temporal dynamics analyses converged, showing greater reappraisal-related neural responses in HC, compared to SAD, during the late reappraisal period. In the one previous paper that examined the temporal dynamics of neural responses during reappraisal, findings indicated greater reappraisal-related neural responses in HC, compared to HC, during the early reappraisal period [3]. That study utilized one of the three tasks reported in the current paper (negative self-beliefs) and tested reappraisal processes using similar reappraisal training methods. However, while the current study focused on between-group effects of the contrast of reappraise *versus* react, separately in the early and late responses, the goal of the previous study was to examine linear decreases in emotional reactivity and increases in regulatory responses during the whole 9-s trial. Thus, the previous study used linear regression to examine linear changes in BOLD responses over time, and compared early *versus* late BOLD responses on each trial, separately for reappraise and for react, in SAD compared to HC. In the present study, we ran between-group t-tests for early and late responses separately, but contrasted the reappraise and react conditions. In addition to these differences in data analytic approaches, the previous study examined one task, while three different contexts were tested in the current paper. Despite these dissimilarities, the results of both studies clearly suggest different timing of the brain responses in SAD and HC during reappraisal. The idea that differences in the temporal dynamics of the brain response are a key factor in regulation processes is consistent with recent findings by Goldin and colleagues, who showed changes in the timing of the BOLD responses in patients with SAD following an 8-week mindfulness-based stress reduction program, and following 16 sessions of individual CBT for SAD [14].

### Task independent reappraisal responses

For each task separately, examination of reappraisal-related BOLD responses common to patients and HC revealed a task-specific network of regions. These regions are implicated in visual attention (cuneus, precuneus, lingual gyrus), working memory (dorsolateral PFC), cognitive regulation (dorsomedial PFC), memory (hippocampus), and language (left MTG, left precentral gyrus) processes, which all take part during reappraisal. Of these regions, the dorsomedial PFC was the only region showing increased activity for both HC and patients during all three tasks.

The DMPFC has been implicated in multiple cognitive functions, including strategic evaluation, introspection, and decision-making [38-40]. In the present study, though no group differences in DMPFC BOLD signal magnitude were found, time course analyses revealed increased late DMPFC activity in healthy controls, compared to SAD patients, when reappraising negative self-beliefs. This finding accords well with the study by Bruhl and colleagues [11], which found comparable MPFC activity in SAD patients who applied reality-checking to negative stimuli and in SAD patients who just perceived the stimuli with no regulation attempts. The researchers suggested that the lack of increased recruitment of MPFC activity due to cognitive control might point to deficits in emotion regulation processes in SAD.

This idea that the lack of additional recruitment of MPFC could be an important neural correlate of emotion regulation deficits in SAD is supported by the results of the current study, and more specifically by the convergence between the neural and behavioral findings: compared to SAD, HC manifested both greater late DMPFC activity, and greater percent reduction in negative emotion ratings, when reappraising negative self-beliefs. In addition, in patients with SAD, greater late DMPFC responses were associated with greater reduction in negative emotion ratings. Thus, decreased DMPFC activity in SAD might be associated with reduced emotion regulation capability, and consequently to reduced ability to down regulate negative reactivity.

## Conclusions

The present study found reduced late BOLD responses in PFC regions in SAD, compared to healthy controls, when reappraising harsh faces. In addition, reduced late responses in the DMPFC in patients with SAD, compared to controls, were related to less reduction in negative emotion ratings when reappraising negative self-beliefs. Together, these results suggest deficient cognitive reappraisal processes in SAD. Importantly, these results indicate that probes with different stimulus dimensions (visual/linguistic, static/dynamic, general/idiographic) are associated with different reappraisal-related behavioral and brain responses. While reappraisal of faces was associated with increased prefrontal activity in HC when compared to patients with SAD, but with no between-group behavioral effects, reappraisal of beliefs was associated with less ability to down regulate negative emotions in patients, compared to HC, with much less robust between-group neural differences. It is important for future research to specifically examine which of these stimulus dimensions could be the most informative in studying reappraisal processes in SAD.

The results of this study suggest that when cued, patients with SAD do try to implement cognitive reappraisal, but their efforts are less efficient, leading to less than optimal emotional relief. Though reappraisal training is a crucial part of CBT for SAD, our results emphasize the importance of teaching patients how to improve the effectiveness of their reappraisal efforts. Mastering more adaptive regulation processes will help patients with SAD reduce the negative emotions they experience.

An interesting question arising from this study is whether, when no external cue exists, patients use cognitive reappraisal less frequently than healthy controls. To answer this question, future studies may examine the extent to which patients with SAD activate un-cued implicit emotion regulation, compared to healthy controls, in addition to examining the associated brain regions that are activated during implicit, *versus* explicit, emotion regulation processes.

One possible limitation of the current study is the specificity of the stimuli that were chosen to evoke negative emotional response. Because of the social nature of these stimuli, these stimuli were probably more emotionally evocative for patients than for HC. Future studies could examine whether regulatory deficits in SAD, behaviorally and in the brain, are specific to socially-related stimuli, or whether this is a more general deficit.

Finally, the current findings stress the importance of performing analyses that elucidate neural temporal change. Here we focused on temporal dynamics of cognitive reappraisal. However, when a patient with SAD enters a social situation, many other regulatory processes such as rumination, attention deployment, and expression suppression are activated. Although the theory suggests that distinct forms of emotion regulation have their own neural circuitry and temporal features [41], in SAD, the temporal dynamics of the BOLD response in regulatory brain regions are still not well understood. Future studies could examine brain activity related to different regulatory processes, taking place at different points in the emotion-generative process.

## Endnote

^a^The increased reappraisal-related activity during criticism was found in two clusters identified as the left fusiform gyrus (1. x,y,z = -24, -68, -12, voxel size = 16; 2. x,y,z = -45, -65, -16, voxel size = 6) and in three clusters identified as the right cerebellum (1. x,y,z = 3, -68, -9, voxel size = 12; 2. x,y,z = 24, -51, -16, voxel size = 7; 3. x,y,z = 17, -58, -12, voxel size = 7). All demonstrated similar pattern of temporal dynamics. For simplicity, we report here only the effects of the biggest cluster in each region (16 voxels for the left fusiform, 12 voxels for the right cerebellum).

## Competing interests

The authors declare that they have no competing interests.

## Authors’ contributions

MZ participated in the study’s design and coordination, contributed to data acquisition, conducted the fMRI sessions, conducted the data analyses, and took the lead on writing the manuscript. PRG helped conceive of the design of the study, participated in the study’s design and coordination, contributed to data acquisition, conducted the fMRI sessions, consulted on data analyses, and contributed to writing the manuscript. HJ participated in the study’s design and coordination, contributed to data acquisition, conducted the fMRI sessions, consulted on data analyses, and contributed to writing the manuscript. KSH consulted on data analyses, and contributed to writing the manuscript. JJG helped conceive of the design of the study, consulted on data analyses, and contributed to writing the manuscript. All authors have read and approved the final manuscript.

## Supplementary Material

Additional file 1: Figure S1 Negative emotion ratings in patients with SAD and in HC when reacting to and reappraising Faces (a), Criticism (b), and Beliefs (c). Left - Negative emotion ratings during the react and reappraise conditions. Right - Percent reduction in negative emotion ratings following reappraisal. Click here for file
