# Supplementary figures and images for: Emotion regulation in social anxiety disorder: behavioral and neural responses to three socio-emotional tasks
Source: Biol Mood Anxiety Disord. 2013 Nov 4;3:20. doi: 10.1186/2045-5380-3-20 (PMC4029608; doi:10.1186/2045-5380-3-20)

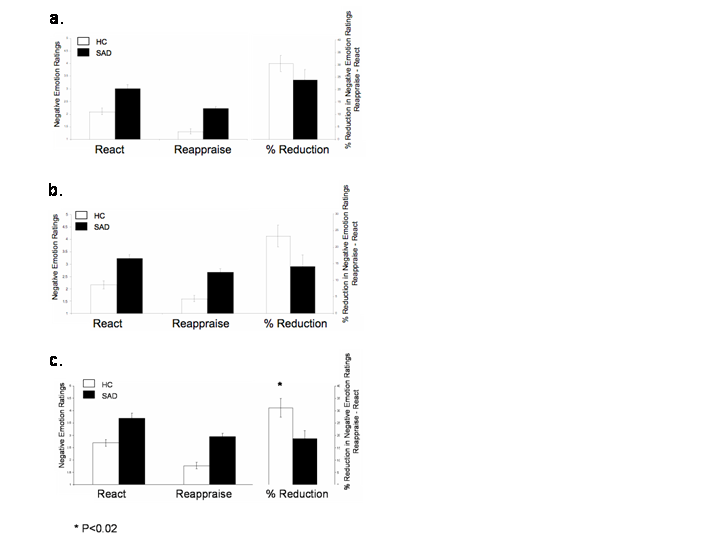

Supplement: Additional file 1: Figure S1 — Negative emotion ratings in patients with SAD and in HC when reacting to and reappraising Faces (a), Criticism (b), and Beliefs (c). Left - Negative emotion ratings during the react and reappraise conditions. Right - Percent reduction in negative emotion ratings following reappraisal. [file 2045-5380-3-20-S1.png]
